# Solid-State Fermented Discarded Dates as a Functional Feed Ingredient: Effects on Meat Quality, Fatty Acid Profile, and Essential Amino Acid Composition

**DOI:** 10.3390/vetsci13070641

**Published:** 2026-06-30

**Authors:** Ali Mujtaba Shah, Dongxu Xia, Wence Wang, Yuan Yuan, Ali Raza Shah, Ali Mustafa Shah, Nazir Ahmed Khan, Weijie Pan, Wei Shi, Guoqiang Chen, Fu Yang, Hongxia Zhao, Qingyun Cao

**Affiliations:** 1 State Key Laboratory of Swine and Poultry Breeding Industry, College of Animal Science, South China Agricultural University, Guangzhou 510642, China; alimujtabashah@sbbuvas.edu.pk (A.M.S.); wangwence@scau.edu.cn (W.W.); chenguoqiang3248@163.com (G.C.); 18156908253@163.com (F.Y.); 2 Inner Mongolia Key Laboratory of Veterinary Fundamentals and Disease Prevention and Control for Herbivorous Livestock, College of Veterinary Medicine, Inner Mongolia Agricultural University, Hohhot 010018, China; xia19861608693@163.com (D.X.); pwj1976933477@163.com (W.P.); hongyuan345@gmail.com (W.S.); 3 Chongqing Three Gorges Vocational College, Wanzhou, Chongqing 404100, China; 2008060033@cqsxzy.edu.cn; 4 Khairpur College of Agriculture and Management Sciences, Sindh Agriculture University, Tandojam 07005, Pakistan; arshah@sau.edu.pk; 5 Benazir Bhutto Shaheed University of Technology and Skill Development, Khairpur Mirs 66020, Pakistan; mustafa@bbsutsd.edu.pk; 6 Department of Animal Nutrition, The University of Agriculture, Peshawar 25130, Pakistan; nazir.khan@aup.edu.pk

**Keywords:** solid-state fermentation, discarded dates, agricultural waste valorization, goat nutrition, growth performance, meat quality, antioxidant status

## Abstract

Palm fruits are produced extensively in tropical and subtropical regions and consumed worldwide. However, over 20% of the total yield is discarded due to inferior quality, resulting in significant agricultural waste and economic loss. To mitigate this challenge and enable the safe valorization of discarded dates (DD) in animal feeding systems, this study employed solid-state fermentation (SSF) to upgrade the nutritional quality of DD and evaluated its potential as a functional feed ingredient for goats. Twenty-four male goats (6 months old; initial body weight 25.86 ± 0.25 kg) were randomly assigned to one of three dietary treatments: a basal diet (control), a diet containing 10% raw DD (D1), and a diet containing 10% solid-state fermented DD (D2). Inclusion of DD in the diet significantly increased average daily gain (ADG), final body weight (BW), and feed efficiency, with the highest values recorded for D2 (*p* < 0.05). Feeding of DD altered (*p* < 0.001) all measured rumen fermentation parameters, except pH, with higher levels (*p* < 0.05) of total volatile fatty acids, propionate, microbial crude protein, and ammonia nitrogen recorded for D1 and D2, as compared to control. Similarly, blood biochemistry revealed elevated total protein, albumin, and globulin in both supplemented groups (*p* < 0.05), whereas higher glucose and cholesterol levels were recorded for the D1 group (*p* < 0.05). Notably, systemic antioxidant status improved with the inclusion of SSF fermented DD, as evidenced by increased superoxide dismutase, glutathione peroxidase, and catalase activities, alongside reduced malondialdehyde levels (*p* < 0.05). The inclusion of DD in the diet decreased cooking and drip losses, and decreased shear force (indicating enhanced tenderness) and water-holding capacity (*p* < 0.05), with better values recorded for D2. Carcass protein and fat contents increased with the inclusion of DD in the diet, with higher values recorded for D2 (*p* < 0.05). Fatty acid analysis revealed higher (*p* < 0.05) contents of rumenic acid and octadecenoic acid in D2, as compared to D1 and control. The concentrations of lysine, methionine, threonine, leucine, and valine in meat were also higher in D2-fed goats (*p* < 0.05). In conclusion, incorporating solid-state-fermented discarded dates into goat diets represents a promising and sustainable strategy to valorize agricultural waste while concurrently improving growth performance, antioxidant status, meat quality, and selected nutrient profiles of goat meat. These preliminary findings warrant validation in larger-scale production.

## Simple Summary

Each year, over 20% of the global date-fruit harvest is discarded due to inferior quality, resulting in significant agricultural waste and economic losses. This study investigated the potential of solid-state fermentation to transform discarded dates into a safe and nutritionally enriched feed ingredient for goats. The fermentation process increased the contents of crude protein by 35.3% and total phenols by 46.8%. Feeding of fermented dates to goats at 10% of the diet resulted in significant improvement in rumen fermentation, growth performance, and systemic antioxidant status compared to raw dates. However, ruminal pH decreased substantially (from 6.72 to 5.57), which warrants further investigation. Meat from goats fed with fermented dates exhibited better tenderness, higher levels of essential amino acids (lysine, methionine), and rumenic acid. These preliminary findings suggest that solid-state fermentation represents a promising approach for valorizing agricultural waste, though economic and environmental assessments are needed to confirm sustainability.

## 1. Introduction

Goats are an important livestock species in tropical and subtropical regions, owing to their ecological adaptability to consume low-quality marginal feed resources and convert them into meat, milk and fiber with superior feed conversion efficiency [1,2]. Goat meat is valued for its high protein and vitamins, and lower cholesterol content compared to pork and chicken [3,4,5]. With improving living standards, demand for goat meat in China has steadily increased. However, the rapid increase in feed costs represents a major constraint to long-term sustainability of goat production under intensive production systems. This has driven interest in exploration of alternative, locally available feed resources, such as discarded dates (DD).

Discarded dates serve as a valuable, energy-dense (13.1 MJ/kg dry matter (DM) metabolizable energy (ME)) feed ingredient in ruminant diets, offering readily fermentable sugars, essential minerals like potassium and magnesium, and bioactive compounds [6]. Inclusion of DD in ruminant diets can reduce feed costs, valorize abundant by-products, and improve palatability, energy density, animal performance, and the beneficial fatty acid profile of meat and milk. DD are also a rich source of vitamin C, B-complex vitamins, and antioxidants [7]. A significant number of regions worldwide lack access to cost-effective feedstuffs for animal production, resulting in dependency on imported feeds [8]. In addition, rapidly rising feed ingredient prices due to competition for grains and other concentrate ingredients across food, feed, and fuel sectors further exacerbate this predicament [9] and increase demand for the development of alternative, low-cost, sustainable feed resources.

Despite great potential, the practical application of DD in animal feeding faces several constraints, including variability in the composition (moisture, nutrient composition, and phenolic profiles) due to differences in varieties, growing conditions, and storage duration. Storage stability of raw dates is also limited due to their high sugar content, which promotes microbial spoilage and fermentation. Furthermore, the economic feasibility and scalability of fermentation processes for large-scale application in animal feeding operations require careful evaluation, including considerations of infrastructure, energy costs, and consistent product quality.

Previous studies have evaluated the application of DD in ruminant nutrition, primarily focusing on their use as energy substitutes in concentrate diets. For instance, Khattab and Anele (2022) reported that replacing barley grain with DD in sheep diets maintained dry matter intake and digestibility while reducing feed costs [8]. Similarly, Berrighi and Aslan (2025) demonstrated that DD could replace concentrate in lamb diets without compromising growth performance or meat quality [10]. However, these studies have primarily used DD, and the potential of fermentation to enhance the nutritional value of DD remains underexplored. Furthermore, previous research has not systematically evaluated the effects of fermented dates on meat quality, fatty acid profile, and essential amino acid composition in goats.

Global agricultural systems face the dual challenge of enhancing food security while managing immense volumes of by-products and non-marketable produce, the disposal of which poses significant economic and environmental burdens [11,12,13]. This is particularly pertinent in date-producing regions, where a substantial proportion of the annual yield—comprising undersized, damaged, overripe, or otherwise non-edible dates—is often relegated to waste or low-value applications, representing a critical loss of potential nutrients and economic value. The valorization of agricultural residues through biotechnological processes, such as solid-state fermentation (SSF), is a promising strategy [11,14]. However, a significant knowledge gap exists regarding the application of SSF-treated DD in ruminant nutrition, as previous studies have primarily focused on raw date products [7,8]. SSF has been shown to enhance the nutritional value of various agricultural substrates by increasing crude protein (CP), reducing anti-nutritional factors (ANFs), and generating bioactive compounds [15,16]. However, the effectiveness of SSF depends on substrate characteristics, microbial strains, and fermentation conditions, requiring case-specific optimization. While SSF has been successfully applied to various agro-industrial wastes for animal feeding, its specific optimization for DD, a uniquely high-sugar, readily fermentable resource, remains underexplored. Transforming DD into a functional feed ingredient could not only mitigate environmental impact but also contribute to a circular bioeconomy by improving resource efficiency [16].

Therefore, this study was designed to develop and characterize a nutrient-enriched feed ingredient via engineered SSF of non-edible dates, using a consortium of probiotic fungi, yeast, and lactic acid bacteria. While previous studies have used raw DD as a partial grain replacement, research on the utilization of fermented DD in ruminant nutrition remains limited. This study contributes to the growing body of evidence by (1) characterizing nutritional and bioactive properties of the multi-strain solid-state fermented DD; (2) evaluating effects on growth performance, rumen fermentation, blood biochemistry, and meat quality; and (3) assessing fatty acid and amino acid profiles of meat, following feeding of SSF-treated dates to goats. This study provides preliminary data on SSF’s potential to transform an underutilized waste stream into a functional feed ingredient, thereby contributing to sustainable agricultural waste management strategies.

## 2. Materials and Methods

### 2.1. Animal Ethical Approval

The current research was conducted in accordance with the rules and regulations of the College of Veterinary Medicine, Inner Mongolia Agricultural University, and all methods involving animals were reviewed and approved by the ethical committee under protocol number NND2023125.

### 2.2. Design of the Animal Experiment and Diet Preparation

The trial was conducted on a goat farm located in Hohhot, Inner Mongolia, China. A total of twenty-four 6-month-old (body weight (BW) = 25.86 ± 0.25 kg) male goats were randomly assigned to one of the three dietary treatments. The three isonitrogenous and isoenergetic dietary treatments were: (1) basal diet without discarded dates (control); (2) basal diet plus 10% discarded dates (D1); (3) basal diet plus solid-state fermented discarded dates 10% (D2).

The 10% inclusion level was selected based on previous studies showing that up to 10% discarded dates can be included in ruminant diets without adversely affecting feed intake [17], whereas levels exceeding 15% may reduce palatability and ruminal pH [18]. This level also aligns with recommendations for high-sugar by-products in ruminant nutrition [8]. The experimental period lasted for 90 days, preceded by a 14-day adaptation phase during which antiparasitic treatment was administered to ensure good health.

Goats were housed in individual pens (3 m^2^, raised floors) equipped with feed and water troughs in the same shed. Sample size (*n* = 8 per treatment) was determined using G*Power software (SPSS v. 26.0 (Chicago, IL, USA) based on an expected effect size of 0.40 for average daily gain (ADG; derived from a pilot study with 4 goats per treatment), α = 0.05, and power = 0.80, which required a minimum of 7 goats per group. Eight goats per treatment were enrolled to account for potential attrition.

Goats had free access to water and feed. Feed was provided twice daily at 08:00 and 18:00. The basal total mixed ration (TMR) was formulated according to AFRC (1993) and NRC (2007) recommendations, using the Cornell Net Carbohydrate and Protein Systems (CNCPS v6.5). Discarded dates and SSFDD were incorporated into the basal diet by replacing wheat bran (reduced from 15% to 5%) and a portion of corn (reduced from 36.5% to 31.5%), while rice straw and silage corn remained constant at 15% each to maintain consistent roughage intake. All experimental diets were formulated to be isonitrogenous and isoenergetic; the numerical differences in CP (11.0–11.4%) and ME (10.12–10.29 MJ/kg DM) fall within the acceptable analytical variation of feed analyses (<3%). The chemical composition of the basal diet is presented in Table 1.

### 2.3. Date Solid-State Fermentation

Fresh, pitted discarded dates were chopped (5–10 mm), mixed with 10% wheat bran, and adjusted to 50–51% moisture. The substrate was pasteurized at 85–90 °C for 30 min, cooled, and inoculated with a mixed culture of *Aspergillus oryzae* (MTCC 1846), *Saccharomyces cerevisiae* (MTCC 170), and *Lactobacillus plantarum* (MTCC 1407) at 10^8^ CFU/g. The inoculated substrate was incubated at 30 ± 1 °C and 70–71% relative humidity for 72 h in perforated trays, with manual turning at 24 h intervals. The fermented product was dried at 50 ± 2 °C for 24 h, ground to pass through a 3 mm sieve, and vacuum-packed until use. A detailed step-by-step protocol is provided in Supplementary File S1. The composition of the DD before and after SSF is presented in Table 2.

### 2.4. Collection of Diet Samples and Chemical Analysis

All feed samples were collected during the experiment, dried at 65 °C for 48 h, and ground to pass through a 0.9 mm screen before analysis. The Association of Official Analytical Chemists (AOAC) and Cornell Net Carbohydrate and Protein System (CNCPS) methods were used for analysis of the samples [18,19]. DM content was determined by drying the samples at 135 °C till constant weight (AOAC method 930.15), CP by the Kjeldahl procedure (AOAC method 984.13), and ash by combustion of the samples at 550 °C for 6 h (AOAC method 942.05). The samples were also analyzed for calcium (AOAC method 984.27), phosphorus (AOAC method 965.17), and ether extract (AOAC method 960.39). The protocol of Van Soest et al. (1991) was followed to determine the contents of acid detergent fiber (ADF; AOAC method 973.18) and neutral detergent fiber (NDF), using heat-stable α-amylase and sodium sulfite as described by Van Soest et al. (1991) [20]. Total phenolic contents were measured using the Folin–Ciocalteu method [21]. Total flavonoids were analyzed via aluminum chloride colorimetry and expressed as mg quercetin equivalents (QE)/g DM [22]. Condensed tannins were analyzed using the vanillin-HCl method [23]. Microbial enumeration involved serial dilution and plating on selective media: MRS agar for lactic acid bacteria (37 °C, 48 h, anaerobic) and PDA for yeast/mold (28 °C, 72 h, aerobic). Mycotoxins (aflatoxin B1, ochratoxin A) were analyzed by ELISA (Romer Labs, Tulln an der Donau, Austria), with detection limits of 2.0 μg/kg and 1.0 μg/kg, respectively. Method validation included spiked recovery experiments (recoveries: 85–110%, CV < 10%) and standard curve linearity (r^2^ > 0.99). Mycotoxin concentrations in both raw and SSF-treated dates were below detectable limits or well below regulatory maximum levels for animal feed (EU Directive 2002/32/EC: aflatoxin B1 < 20 μg/kg for feed materials).

### 2.5. Growth Performance

After the 14-day adaptation period, body weight was recorded on two consecutive days prior to the morning feeding at days 0, 30, 60, and 90. The following calculations were used for average daily gain (ADG), average daily feed intake (ADFI), and feed conversion ratio (FCR):

ADG (Kg/d) = (Final BW − Initial BW)/90 days

ADFI (g/d) = (Total feed offered (g) − Total feed refused (g))/90 days

FCR = ADFI (kg/d)/ADG (kg/d)

### 2.6. Blood Sampling and Biochemical Measurements

On the final experimental day, approximately 4 mL of blood was drawn from the jugular vein of each goat at 08:00 h, before slaughter. Samples were collected into EDTA-containing tubes and transported immediately to the laboratory. To separate plasma, tubes were centrifuged at 3000× *g* for 15 min at 4 °C. The recovered plasma was stored at −20 °C until assays were performed. Plasma concentration of total protein (TP), albumin (ALB), globulin (GLOB; calculated as TP-ALB), glucose (GLU), cholesterol (CHO), triglycerides (TG), and creatinine (CREA) were measured using an automated biochemical analyzer (Shenzhen Mindray Bio-Medical Electronics Co., Ltd BS-480, Shenzhen, China). Commercial kits were used following manufacturer’s instructions. The antioxidant enzyme activities were determined using commercial kits (Nanjing Jiancheng Bioengineering Institute, China): superoxide dismutase (SOD, catalog no. A001-3, WST-1 method), glutathione peroxidase (GSH-Px, catalog no. A005-1, colorimetric method), catalase (CAT, catalog no. A007-1-1, ammonium molybdate method), and malondialdehyde (MDA, catalog no. A003-1, TBA method). All assays were performed on a microplate reader (BioTek Epoch 2, Winooski, VT, USA) at the wavelength specified by each kit. A standard curve was generated using the provided standard (r^2^ > 0.995 for all assays). Intra-assay CVs were <6% and <10%.

### 2.7. Rumen Fermentation

On the last day of the experiment, all animals were stunned using a captive bolt pistol to slaughter, followed by exsanguination in accordance with standard slaughter practices and animal welfare guidelines, and rumen contents were sampled without delay. The pH of the rumen fluid was measured immediately with a digital pH meter (D87 HRB, Tokyo, Japan). The fluid was then passed through four layers of gauze and centrifuged at 1200× *g* for 15 min to remove particulate matter. An aliquot of the clarified rumen fluid was acidified (perchlorate-treated) and later neutralized with potassium hydroxide. Subsequently, the preparations were centrifuged at 400× *g* for 12 min. Microbial crude protein (MCP) was estimated using the purine base method [24]. Rumen fluid (50 mL) was strained through four layers of cheesecloth, then centrifuged at 500× *g* for 10 min at 4 °C to remove feed particles and protozoa. The supernatant was centrifuged at 20,000× *g* for 20 min at 4 °C to pellet microbial cells. The pellet was washed twice with cold saline (0.9% NaCl), with each wash followed by centrifugation at 20,000× *g* for 15 min. The final pellet was hydrolyzed in 0.25 M H_2_SO_4_ at 100 °C for 2 h, and purine bases (adenine, guanine) were quantified by HPLC (Agilent C18 column, Santa Clara, CA, USA, UV 260 nm). MCP was calculated as MCP (mg/dL) = (purine-N × 70)/0.8 [24], and ammonia nitrogen (NH_3_-N) was determined using the phenol–hypochlorite colorimetric method as described by Broderick and Kang [25]. Volatile fatty acids were quantified using a high-performance liquid chromatography system as described by Shah et al. [26].

### 2.8. Meat Samples Collection and Analysis

Immediately after slaughtering, carcass samples were taken from the *longissimus thoracis* muscle between the 12th and 13th ribs on the right side and stored at 4 °C until analysis. Meat quality traits were evaluated as follows. Moisture content was determined by drying the samples in an oven at 102 °C (method 950.46) until a constant weight, CP (N × 6.25) by Kjeldahl procedure (method 978.04), ash by incinerating the meat samples in a muffle furnace (920.153), and ether extract by Behr Labor-Technik GmbH, Düsseldorf, Germany (method 930.09), using petroleum ether as the extraction solvent [27]. The meat color was determined using a portable colorimeter (Model WR-10, Shenzhen Wave Optoelectronics Technology Co., Ltd., China) with an 8 mm viewing aperture, D65 illuminant, and 10° standard observed. The instrument was calibrated using a standard white plate (X = 94.46, Y = 100.10, Z = 107.66) provided by the manufacturer before measurements. The color values are displayed as lightness (L*), redness (a*), and yellowness (b*). Cooking loss was measured as weight loss by weighing the muscle samples after heating and cooling the samples at 75 °C in a water bath. The shear force was measured (using TMS-PRO (FTC Co., Sterling, VA, USA)) at a speed of 200 mm/min, cutting the long strip of meat (0.5 × 0.5 × 0.5 cm^3^) along the muscle fibers after cooking the samples. Fatty acid methyl esters (FAMEs) were separated and quantified using a gas chromatograph (Agilent 7890A, Santa Clara, CA, USA) equipped with a flame ionized detector and an HP-88 capillary column (100 m × 0.25 mm × 0.20 µm film thickness). Helium served as the carrier gas at 1.2 mL/min. The oven temperature program was as follows: initial 100 °C held for 5 min, increased to 240 °C at 4 °C/min, held for 15 min. Injection and detector temperatures were 250 °C and 280 °C, respectively. FAMEs were identified by comparison with an external standard (37-component FAME mix, Supelco 47886-U; individual CLA isomers, Matreya LLC, Pleasant Gap, PA, USA, catalog nos. 1255 and 1256). Quantification used calibration curves (5–200 µg/mL, r^2^ > 0.99). A control standard was injected every 10 samples; CVs for retention time were <0.5% and for peak area were <5%. Amino acids were quantified by HPLC (Agilent 1260 infinity) with pre-column OPA/FMOC derivatization, separated on a Zorbax Eclipse Plus C18 column (Agilent Technologies, Santa Clara, CA, USA) (4.6 × 100 mm, 3.5 µm) using a gradient mobile phase at 1.5 mL/min and 40 °C, and detected by fluorescence (340/450 nm for OPA; 266/305 nm for FMOC). Standards (Sigma-Aldrich AAS18, St. Louis, MI, USA) were used for quantification.

### 2.9. Statistical Analysis

All data were initially organized in Microsoft Excel (version 2016), and statistical evaluations were then conducted using SPSS v. 26.0 (Chicago, IL, USA). Data distribution normality was assessed using the Shapiro–Wilk test, and homogeneity of variance was tested using Levene’s test. All outcomes met the assumption of normality (*p* > 0.05) and homogeneity of variance (*p* > 0.05). Body weight data were analyzed using a repeated-measures mixed model with treatment, time, and their interaction as fixed effects, animal as a random effect, using autoregressive covariance, and Tukey’s adjustment. A one-way analysis of variance (ANOVA) was applied to test the effect of dietary treatments (three levels: control, D1, and D2) on each outcome variable. When overall treatment effects were detected (*p* < 0.05), Tukey’s Honestly Significant Difference (HSD) test was used for pairwise multiple comparisons. Graphs and other visual outputs were generated with GraphPad Prism version 8.0.2. Results are presented as mean ± SEM.

## 3. Results

### 3.1. Growth Performance

The results of the growth performance are presented in Table 3. Compared to the control, inclusion of both untreated DD and SSF treated DD (SSFDD) significantly increased (*p* < 0.05) the BW of the goats on d 30, 60, and 90. Further comparisons revealed that the SSFDD supported greater (*p* < 0.05) BW at day 30 (34.47 vs. 33.09 kg), 60 (44.00 vs. 41.64 kg), and 90 (52.04 vs. 46.15 kg) compared to untreated DD. Moreover, supplementation with DD and SSFDD significantly increased ADG, ADFI, and feed efficiency compared with the control group (*p* < 0.05). Notably, goats fed with SSFDD had greater (*p* < 0.05) ADG (0.58 vs. 0.51 kg/d) and FCR (4.84 vs. 6.72) than the untreated DD group.

### 3.2. Rumen Fermentation

Table 4 presents data on the effect of untreated DD and SSFDD on rumen fermentation characteristics of goats. The inclusion of untreated DD and SSFDD significantly increased the contents of propionate, TVFA, MCP, and NH_3_-N compared to the control group (*p* < 0.05). However, untreated DD and SSFDD inclusion in the diets significantly reduced pH and acetate compared to the control group (*p* < 0.05). The observed pH values in date-supplemented groups (5.57–5.75) approach the threshold for subacute ruminal acidosis (pH 5.5–5.6), though no clinical signs of acidosis were observed during the study. In addition, no significant (*p* > 0.05) effect of these supplementations was observed on the butyrate and acetate to propionate ratio. Except for NH_3_-N, which was greater in SSFDD, there were no differences in all measured rumen fermentation parameters between the untreated DD and SSFDD.

### 3.3. Blood Biochemistry

**Figure 1:**
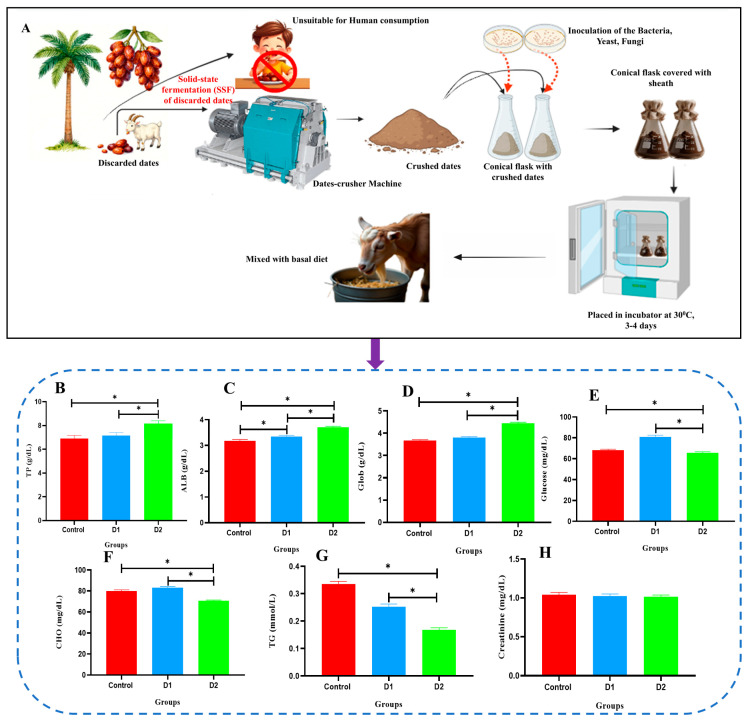
Effect of discarded dates and SSF discarded dates supplementation on blood biochemistry in goats. (**A**) Schematic diagram of the experimental design and fermentation process. (**B**) Total Protein concentrations, (**C**) albumin concentrations, (**D**) globulin concentrations, (**E**) glucose concentrations, (**F**) cholesterol concentrations, (**G**) triglycerides concentrations, (**H**) creatinine concentrations. Asterisks (*) indicate significant differences among treatment groups (*p* < 0.05), (*n* = 8).

The blood biochemistry results of the current research trial are presented in Figure 1A–H. The inclusion of untreated DD and SSFDD significantly increased the concentrations of the TP, ALB, and GLOB compared to the control group (*p* < 0.05). However, untreated DD supplementation increased glucose and CHO levels compared with the SSFDD and control groups. The inclusion of untreated DD and SSFDD in the diet decreased TG levels compared with the control group (*p* < 0.05).

### 3.4. Antioxidant Profile

**Figure 2:**
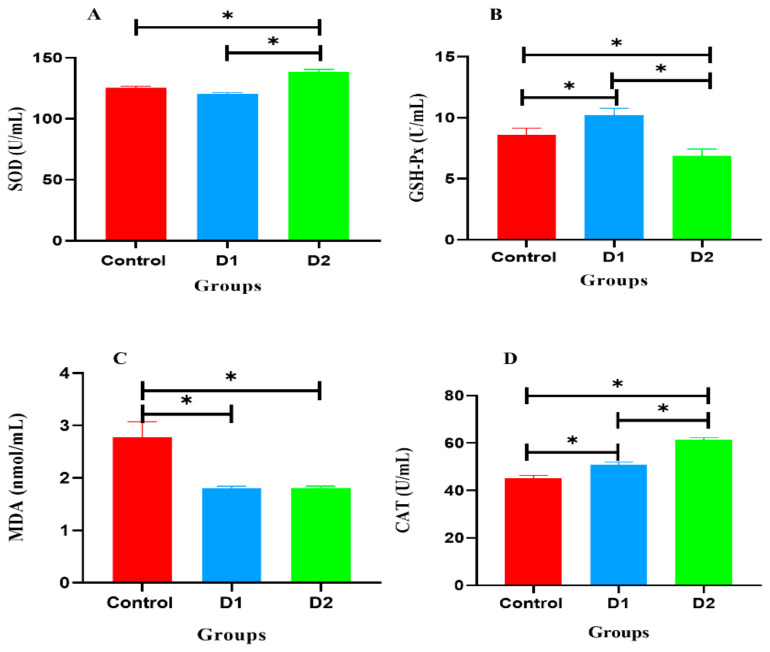
Effect of discarded dates and SSF discarded dates supplementation on antioxidant profile in goats. (**A**) Superoxide dismutase levels, (**B**) glutathione peroxidase levels, (**C**) malondialdehyde levels, (**D**) catalase levels. Asterisks (*) indicate significant differences among treatment groups (*p* < 0.05), (*n* = 8).

Data on the effects of feeding raw and SSFDD on the antioxidant profile of goats are visualized in Figure 2A–D. The inclusion of DD and SSFDD significantly increased SOD, GSH-Px, and CAT levels compared with the control group (*p* < 0.05). However, the inclusion of DD, regardless of treatment, significantly reduced MDA levels compared with the control group (*p* < 0.05). The observed improvements in antioxidant status are consistent with enhanced phenolic bioavailability following SSF. However, the specific molecular pathways (such as Nrf2 activation) were not directly measured in this study and require further investigation. Further comparisons between the treatment groups showed that SOD and CAT levels were higher, whereas GSH-Px was lower in the SSFDD group than in the DD group.

### 3.5. Carcass Composition

Data on the effects of untreated DD and SSFDD supplementation on carcass composition in goats are presented in Table 5. The inclusion of DD, regardless of treatment, significantly increased the carcass protein and fat percentages compared to the control group (*p* < 0.05). Further comparison among the treatment groups revealed that the greatest increase in carcass protein and fat percentage was observed in goats fed SSFDD (*p* < 0.05). The moisture content decreased numerically but not significantly (*p* = 0.142), while ash remained unchanged across all groups (*p* = 0.344).

### 3.6. Meat Quality

Table 6 summarizes data on the effect of untreated DD and SSFDD supplementation on meat quality in goats. Feeding DD, regardless of treatment, significantly increased WHC and decreased cooking loss, drip loss, and shear force (*p* < 0.001), indicating improved tenderness. Among the treatments, SSFDD showed the greatest increase in WHC (*p* < 0.05) and the greatest decreases in cooking loss, drip loss, and shear force (*p* < 0.05). Compared with the control, color parameters were also affected; lightness (L*) and redness (a*) increased significantly with the inclusion of DD in the diet, while yellowness (b*) and pH at 24 h showed no significant differences among groups (*p* > 0.05).

### 3.7. Fatty Acid Profile

Data on the effect of feeding untreated DD and SSFDD on carcass fatty acid profile are presented in Table 7. Inclusion of the untreated DD and SSFDD significantly increased the levels of functional fatty acids, octadecenoic and rumenic acids compared to the control group (*p* < 0.05). Notably, the meat of the SSFDD-fed goats had higher concentrations of octadecenoic and rumenic acids than the untreated DD group. Moreover, the inclusion of dates, regardless of treatment, significantly reduced the levels of palmitoleic and linoleic acids compared with the control group (*p* < 0.05).

### 3.8. Amino Acid Profile

Table 8 summarizes data on the changes in the carcass amino acid profile of goats in response to feeding of untreated DD and SSFDD. Inclusion of untreated DD and SSFDD in the diet significantly increased the concentrations of lysine, methionine, threonine, leucine, and valine (*p* < 0.05) and decreased glutamic acid concentration compared with the control group (*p* < 0.05). Comparison between SSF-treated and untreated DD showed that SSFDD had higher concentrations of lysine, methionine, threonine, leucine, and valine than the untreated DD-fed group (*p* < 0.05). No significant differences were detected for the other amino acids measured in the carcasses of goats (*p* > 0.05).

## 4. Discussion

The findings of the present study revealed that SSF treatment improves the nutritional value of DD (unacceptable for human consumption), and SSFDD can be efficiently used as a feed ingredient in goat diets. However, the present study provides preliminary evidence in a small-scale trial, and further investigations are required to evaluate its economic feasibility, long-term effects on animal health and productivity, and optimal inclusion levels in diverse production systems. Our findings highlight the huge scope of efficiently utilizing SSF-treated dates in goat rations in the tropics. Date palms, already adapted to arid and semi-arid climates of the tropics, produce nutrient-dense fruits and generate significant agricultural waste, including DD [28]. During dry periods when green fodder is unavailable, DD-based feed can provide goats with essential energy [29,30]. In addition, by using SSF techniques to treat DD, we can improve its nutritional quality and reduce the levels of toxins and anti-nutritional factors.

In the present study, the SSF process significantly increased CP content of DD from 6.8% to 9.2%, representing a 35.3% relative increase (Table 2). This CP enrichment can be associated with two complementary mechanisms, (1) true microbial protein synthesis, wherein *S. cerevisiae*, and *L. plantarum* utilize the readily available sugar in dates as a carbon source to produce fungal and bacterial biomass rich in amino acids; and (2) a concentration effect resulting from the partial catabolism of soluble carbohydrates during fermentation, which reduced total sugar content from 68.5 to 52.3% while preserving or increasing nitrogenous components [29,30]. The synergistic action of this microbial consortium, where *A. oryzae* secretes extracellular proteases and amylases that liberate fermentable substrates, *S. cerevisiae* produces growth-promoting metabolites, and *L. plantarum* lowers pH to create selective conditions, contributes to the observed net protein accretion. This dual mechanism of protein enrichment through SSF aligns with the previous report on the volatilization of sugar-rich agricultural by-products during fermentation [16]. Although the microbial protein content of SSFDD was not directly quantified, the 35.3% CP increase and 23.7% sugar reduction provide indirect evidence of microbial protein synthesis. The improved growth performance and rumen fermentation observed with 10% SSFDD inclusion suggest that the enhanced protein was at least partially bioavailable [31]; however, direct quantification of microbial protein flow to the duodenum would be required for confirmation.

In the current study, we found that the inclusion of DD and SSFDD significantly increased BW on d 30, 60, and 90. In addition, these supplements improved rumen fermentation parameters, including propionate, TVFA, MCP, and NH_3_-N levels, compared with the control group. The improvement in growth performance is associated with enhanced nutrient bioavailability and altered rumen microbial ecology. Raw DD contains high levels of readily fermentable sugars (glucose and fructose), which increase ruminal VFA production, the primary energy source for ruminants [18]. SSF further enhances this effect by degrading anti-nutritional factors, increasing soluble carbohydrate and CP content, and enriching the substrate with microbial enzymes that pre-digest the ration. Fermented substrates create a more favorable rumen environment by selectively stimulating cellulolytic bacteria and protozoa, thereby improving fiber degradation, rumen pH, and the molar proportions of propionate, a glucogenic VFA that promotes lipogenesis and growth [18]. These findings are consistent with studies showing improved growth performance in lambs fed date pits [32,33].

However, the substantial reduction in ruminal pH (from 6.72 in control to 5.57 in SSFDD) warrants careful consideration. While no clinical signs of subacute ruminal acidosis (SARA) were observed, pH values in the SSFDD group approached the threshold (5.5–5.6) associated with SARA in ruminants [34]. The inclusion of sodium bicarbonate (0.4%) in all diets likely provided buffering capacity, but the margin of safety appears narrow. Prolonged pH near 5.5 may have subclinical effects, including reduced fiber digestibility and rumen epithelial damage [31]. The 0.4% sodium bicarbonate provided buffering, but higher levels (0.6–0.8%) or alternative buffers may be warranted in practice. Individual variation in pH response should also be considered. This observation underscores the need for careful management when feeding high-sugar ingredients and suggests that lower inclusion rates or additional buffering agents may be necessary in practical application. Therefore, when fermented discarded dates are included in commercial feeding systems, we recommend routine monitoring of ruminal pH and observation for signs of subacute ruminal acidosis (e.g., reduced feed intake, diarrhea, or lameness), particularly during the adaptation period and when dietary transitions occur.

The increase in glucose observed in the raw date group (D1) likely reflects the high sugar content of untreated dates, which may not be metabolically optimal compared to the fermented product. This is one of the advantages of SSF, which reduces readily fermentable sugars (from 68.5% to 52.3%) and promotes more gradual glucose release. Similarly, elevated cholesterol in the D1 group warrants attention, as it may represent increased de novo lipogenesis driven by sugar availability rather than improved nutritional quality. Glucose and cholesterol remained within the normal range in goats. SSF mitigated these effects by reducing fermentable sugars, promoting more gradual glucose release, and stable homeostasis.

The significantly elevated TP, ALB, and GLOB in goats fed DD and SSFDD reflect a systemic enhancement of protein metabolism and immunity. Increased TP and ALB indicate improved protein anabolism and liver function, respectively [35,36,37]. By providing readily fermentable carbohydrates in DD, the rumen can produce more absorbable amino acids required for albumin synthesis, and amino acids are spared from catabolism [38]. However, the elevated ammonia nitrogen concentrations in supplemented groups require careful interpretation. While increased NH3-N may indicate enhanced proteolysis and microbial activity, it could also reflect a temporary imbalance between ammonia production and microbial uptake. The concurrent increase in MCP suggests that overall nitrogen utilization was improved, but the relationship between ammonia concentration and microbial protein synthesis is complex and warrants further investigation [25]. While MCP values aligned with previous reports [7,8,39], limitations of the purine base method should be acknowledged. Fine feed particles (<1 µm) may co-sediment with microbial cells despite differential centrifugation and washing, potentially overestimating MCP [40,41]. Conversely, particle-associated bacteria may be lost during low-speed centrifugation. To minimize bias, all samples were collected from the ventral rumen sac, which contains more representative microbial populations with less particle contamination [42]. Future studies should consider ^15^N labeling or molecular markers (e.g., 16S rRNA) to complement MCP estimates. While MCP values aligned with previous reports [7,8,39], limitations of the purine base method should be acknowledged. SSF enriches the diet with high-quality fungal single-cell protein, providing a direct source of digestible amino acids that increase the amount of protein available post-ruminally [43]. This is most pronounced in the SSFDD group. Moreover, fibrolytic enzyme production enhances dietary digestibility, helping to retain nitrogen and synthesize proteins. The increased globulin concentration suggests enhanced protein synthesis and potential immunomodulation. However, globulin includes multiple protein fractions beyond immunoglobulins; therefore, direct measurement of specific immune markers (IgG, IgM, IgA) would be required to confirm improved immune status. This represents a limitation of the current study and warrants further investigation [44].

Goats fed with DD and SSFDD showed increased blood antioxidant enzyme activity (SOD, GSH-Px, and CAT) and reduced MDA, a key marker of lipid peroxidation. This improved systemic antioxidant status may be related to SSF, which, via enzymatic activity (tannases, cellulases, β-glucosidases) from A. oryzae, liberates bound phenolics in dates. Total phenolic content increased by 46.8% (from 4.7 to 6.9 mg GAE/g), reflecting the release of free phenolic aglycones with greater bioavailability. Additional contributions may include Maillard reaction products and microbial metabolites (e.g., exopolysaccharides, short-chain fatty acids). Al-Farsi et al. found that DD are rich in phenolic acids and flavonoids, which are capable of scavenging free radicals, and SSF further bioconverts these polyphenols into more bioavailable forms [45]. The resulting increase in protective enzymes (SOD, GSH-Px, CAT) and reduction in plasma MDA provide functional evidence of reduced oxidative stress [46,47]. While these observations are consistent with the hypothesis that SSF-enhanced phenolics may modulate endogenous antioxidant defenses, it is important to note that activation of specific host pathways, such as Nrf2, was not directly measured in this study. Therefore, the precise molecular mechanisms underlying these effects remain speculative and warrant targeted investigation in future studies. These results are consistent with Fodail et al. [46] and Torghabeh et al. [48], who reported that mixed-source mineral supplementation significantly increased glutathione peroxidase activity and total antioxidant capacity in *Baluchi lambs*.

Goats fed SSFDD showed enhanced meat quality, including higher protein and fat contents, reduced cooking loss, and enhanced color attributes, and improved tenderness (as indicated by lower shear force values). The improvement in meat tenderness and water-holding capacity can be explained by two interrelated mechanisms. First, enhanced antioxidant status—attributable to phenolic compounds in dates—likely protects myofibrillar proteins from oxidative modification post-mortem, preserving protein structure and allowing greater proteolysis by calpains and cathepsins for tenderization. Second, altered lipid metabolism (Table 7) suggests increased intramuscular fat deposition and improved membrane integrity, higher octadecenoic acid (C18:1, including oleic acid) and CLA levels, which increase fiber spacing, lubricate the myofibrillar matrix, and reduce drip loss during rigor. Soluble sugars in DD may shift nutrient partitioning toward protein synthesis and intramuscular fat (marbling) formation rather than visceral fat deposition [10]. SSF further enhances this effect by improving nutrient bioavailability and enriching the diet with microbial metabolites. From a commercial perspective, these improvements are meaningful; a 2.37% reduction in cooking loss and a 1.53% increase in WHC represent approximately 8.6% and 5.4% increases, respectively, which could translate into higher retail yield and improved consumer perception of juiciness. Similarly, a 0.30 kg reduction in shear force (8.8%) represents a notable improvement in tenderness that consumers could detect [37]. Together, reduced oxidative damage and improved lipid profiles underpin the superior tenderness and juiciness in meat from goats fed SSFDD.

Following feeding diets containing DD, the observed increases in specific fatty acids, including saturated fatty acids, octadecenoic acid, and rumenic acid, can be associated with three primary mechanisms: rumen biohydrogenation patterns, increased substrate availability, and metabolic shifts. Firstly, dates contain a high concentration of fermentable sugars that contribute to ruminal propionate production through their glycolytic fermentation. In addition to synthesizing and releasing these fatty acids into circulation for tissue uptake, propionate is a primary precursor to hepatic de novo lipogenesis [49]. It has been shown that the increase in rumenic acid (CLA c9,t11), a bioactive fatty acid with health-promoting properties, is a direct indicator of altered rumen biohydrogenation. Research has shown that dates have a unique sugar profile and bioactive compounds (enhanced by fermentation) that inhibit the final step of biohydrogenation, leading to the accumulation of the intermediate trans-11 vaccenic acid (C18:1 t11), which is then converted into CLA in the animal’s tissues via SCD [50]. While the increase in rumenic acid (from 0.024 to 0.052 mg/100 g) represents a 117% relative increase, the absolute concentrations remain relatively low compared to beef and dairy products. Nonetheless, even a modest increase in bioactive fatty acids may have nutritional relevance in populations where goat meat is a primary protein source. The reduction in linoleic acid (from 3.58 to 3.47 mg/100 g) represents a trade-off that may partially counterbalance the beneficial increase in rumenic acid and octadecenoic acids.

Goat meat from the DD and SSFDD groups had higher concentrations of essential amino acids (lysine, methionine, threonine, leucine, and valine). While the increases in essential amino acids were modest in absolute terms (e.g., 1.8–10.9% for lysine, 10.6% for methionine), even small improvements in limiting amino acids can be nutritionally significant, particularly in regions where goat meat serves as the primary protein source. The reduction in glutamic acid observed in supplemented groups may reflect more efficient nitrogen utilization and preferential partitioning toward protein accretion; however, this interpretation remains speculative and requires further investigation through nitrogen balance studies. Lysine and methionine, the limiting amino acids in goat meat, increased. Even modest improvements in limiting amino acids can enhance protein quality scores (PDCAAS, DIAAS). Glutamic acid reduction is minor and clinically insignificant as it is non-essential. Date products have a protein-sparing effect, in which readily available sugars contribute to ruminal energy, nitrogen production, and microbial protein synthesis [31]. As a result, the small intestine receives high-quality microbial proteins rich in essential amino acids. SSF further enriches dates with fungal single-cell proteins directly, high in limiting amino acids such as lysine and threonine. Fibrolytic enzymes produced during fermentation break down fiber, increasing energy availability and improving MCP [51]. Methionine and BCAA levels have increased markedly, indicating enhanced amino acid supply, as well as an increased anabolic state for muscle protein synthesis [39]. This study did not evaluate other anti-nutritional factors (e.g., saponins, phytate), nutrient digestibility, protein fractions, or cellulolytic bacterial populations—areas warranting future investigation.

## 5. Conclusions

This study provides preliminary evidence that solid-state fermentation is an effective approach for valorizing low-quality discarded dates. Incorporating SSFDD at 10% into goat diets altered serum metabolites, enhanced antioxidant enzyme activities, improved rumen fermentation, and enhanced meat quality parameters. The concentration of beneficial fatty acids and essential amino acids in meat increased with the feeding of SSFDD. However, the substantial reduction in ruminal pH and increased ammonia nitrogen concentrations warrant careful consideration in practical application. Due to the small sample size (n = 8 per treatment) and single inclusion level evaluated, these findings should be considered preliminary. Future research should validate these results in large-scale production trials, evaluate dose–response relationships and include economic analyses to assess the commercial viability of SSFDD as a feed ingredient. The scalability of the fermentation process and the consistency of product quality under industrial conditions also require further investigations. Finally, to fully establish the practical relevance of these findings, future research should validate the performance and health effects of SSFDD under commercial farming conditions, where management practices, environmental stressors, and diet formulations differ substantially from controlled experiment setting.

## Figures and Tables

**Table 1:** Total mixed ration composition of the numerous groups (DM%).

Items	Control	D1 (10% Discarded Dates)	D2 (10% SSF Discarded Dates)
Wheat bran	15.0	5.0	5.0
Soybean meal	3.0	3.0	3.0
Canola meal	3.0	3.0	3.0
Corn	36.5	31.5	31.5
Salt	0.5	0.5	0.5
Sodium bicarbonate	0.4	0.4	0.4
Mineral and vitamins ^1^	0.8	0.8	0.8
Calcium hydrophosphate	0.8	0.8	0.8
Rice straw	15.0	15.0	15.0
Silage corn	15.0	15.0	15.0
DDGS	10.0	10.0	10.0
Discarded dates (raw)	0	10	0
SSF dates	0	0	10
Total	100	100	100
Nutritive content			
DM	76.8	76.5	76.2
EE	2.8	2.9	3
CP	11	11.2	11.4
NDF	31	30.8	30.5
ADF	18.5	18.3	18.1
Ash	6.3	6.4	6.5
Total phenolics (mg GAE/g DM)	2.8	4.2	5.1
Total flavonoids (mg QE/g DM)	1.2	2.1	2.8
Condensed tannins (g/kg DM)	0.6	0.8	0.7
ME (MJ/kg DM)	10.12	10.21	10.29

^1^ Provided by kg diet, sulphate 60 mg as Fe source, sulphate 8 mg for Cu, Sulphate 40 mg as Zn, sulphate 40 mg as Mn, chloride 0.2 mg as Co, iodates 0.3 mg as I, Selenite 0.2 mg as Se. DDGS = distillers dried grains; DM = dry matter; EE = ether extract; CP = crude protein; NDF = neutral detergent fiber; ADF = acid detergent fiber.

**Table 2:** Proximate analysis of the dates and solid-state fermented (SSF) dates.

Item	Discarded Dates (D1)	SSF Discarded Dates (D2)
Dry matter %	92.5	90.08
Crude protein %	6.8	9.2
Ether extract %	1.21	1.41
Crude fiber %	11.5	9.82
NDF %	18.4	15.8
ADF %	12.3	10.8
Ash %	3.1	3.5
Total sugars %	68.5	52.3
pH	5.8	4.4
Lactic acid g/kg DM	ND	32.5
Acetic acid g/kg DM	ND	8.2
Total phenolics mg GAE/g	4.7	6.9
Flavonoids (mg QE/g)	2.3	3.8
Tannins g/kg	1.2	0.8
Microbial load		
Lactic acid bacteria (log CFU/g)	3.2 ± 0.4	8.5 ± 0.3
Yeast (log CFU/g)	2.1 ± 0.3	7.9 ± 0.4
Mold (log CFU/g)	1.5 ± 0.2	6.8 ± 0.3
Mycotoxin Analysis		
Aflatoxin B1 (μg/kg)	ND	<2.0
Ochratoxin (μg/kg)	ND	<2.0

SSF = solid-state fermentation, NDF = neutral detergent fiber, ADF = acid detergent fiber, ND = not detected. Total phenolics expressed as mg gallic acid equivalents (GAE)/g dry matter, determined by the Folin–Ciocalteu method. CFU = colony-forming unit.

**Table 3:** Effect of discarded dates supplementation on growth performance in goats.

Item	Treatments			SEM	*p*-Value
	Control	D1	D2		
BW 0 (kg)	26.2	26.1	25.3	0.239	0.247
BW 30 (kg)	31.44 ^c^	33.09 ^b^	34.47 ^a^	0.295	<0.001
BW 60 (kg)	36.15 ^c^	41.64 ^b^	44.00 ^a^	0.7	<0.001
BW 90 (kg)	41.66 ^c^	46.15 ^b^	52.04 ^a^	0.914	<0.001
ADG (kg)	0.46 ^c^	0.51 ^b^	0.58 ^a^	0.01	<0.001
ADFI (g)	1329.88 ^c^	1498.00 ^a^	1438.75 ^b^	16.665	<0.001
FCR (kg)	7.74 ^c^	6.72 ^b^	4.84 ^a^	0.329	<0.001

BW = body weight, ADG = average daily gain, ADFI = average daily feed intake, FCR = feed conversion ratio, D1 = 10% date-fruit, D2 = 10% solid-state-fermented date-fruit, SEM = standard error of mean. ^a^, ^b^, ^c^ values within the same row with different superscript letters are significantly different (*p* < 0.05). *n* = 8 per treatment group.

**Table 4:** Effect of discarded dates supplementation on rumen fermentation in goats.

Item	Treatments			SEM	*p*-Value
	Control	D1	D2		
pH	6.72	5.75	5.57	0.123	<0.001
Acetate %	62.99 ^a^	57.26 ^b^	57.45 ^b^	0.588	<0.001
Propionate %	15.53 ^b^	19.45 ^a^	19.33 ^a^	0.415	<0.001
Butyrate %	5.67	5.83	5.78	0.031	0.108
TVFA mmol/L	89.61 ^b^	92.71 ^a^	93.25 ^a^	0.419	<0.001
MCP mg/dL	6.27 ^c^	6.27 ^b^	7.73 ^a^	0.139	<0.001
NH_3_-N mg/dL	23.02 ^c^	25.30 ^b^	28.49 ^a^	0.483	<0.001
Acetate: Propionate mg/dL	3.46	3.38	3.25	0.436	0.133

D1 = 10% discarded dates, D2 = 10% solid-state-fermented discarded dates fruit, TVFA = total volatile fatty acid, MCP = microbial crude protein, NH_3_-N = ammonia nitrogen; SEM = standard error of mean. ^a^, ^b^, ^c^ values within the same row with different superscript letters are significantly different (*p* < 0.05). n = 8 per treatment group.

**Table 5:** Effect of discarded dates supplementation on carcass composition of the goats.

Item	Treatments			SEM	*p*-Value
	Control	D1	D2		
Moisture (%)	70.60	70.16	70.12	0.109	0.142
Protein (%)	20.71 ^c^	20.98 ^b^	21.53 ^a^	0.081	<0.001
Fat (%)	6.57 ^c^	7.01 ^b^	7.43 ^a^	0.075	<0.001
Ash (%)	1.07	1.04	1.07	0.010	0.344

D1 = 10% discarded dates, D2 = 10% solid-state-fermented discarded dates, SEM = standard error of mean. ^a^, ^b^, ^c^ values within the same row with different superscript letters are significantly different (*p* < 0.05). *n* = 8 per treatment group.

**Table 6:** Meat quality parameters in different goat groups supplemented with discarded dates.

Item	Treatments			SEM	*p*-Value
	Control	D1	D2		
pH (24 h)	5.60	5.56	5.53	0.013	0.079
WHC (%)	59.54 ^c^	62.54 ^b^	64.91 ^a^	0.464	<0.001
Cooking Loss (%)	27.46 ^a^	24.46 ^b^	21.94 ^c^	0.475	<0.001
Drip Loss (%)	5.00 ^a^	4.10 ^b^	3.47 ^c^	0.133	<0.001
Shear Force (kg)	3.40 ^c^	3.25 ^b^	3.10 ^a^	0.123	<0.001
L* (Lightness)	38.29 ^c^	40.09 ^b^	41.59 ^a^	0.287	<0.001
a* (Redness)	12.19 ^c^	12.49 ^b^	12.79 ^a^	0.556	<0.001
b* (Yellowness)	7.12	7.14	7.19	0.040	0.818

D1 = 10% discarded dates, D2 = 10% solid-state-fermented discarded dates, SEM = standard error of mean. ^a^, ^b^, ^c^ values within the same row with different superscript letters are significantly different (*p* < 0.05). *n* = 8 per treatment group.

**Table 7:** Fatty acid levels in the carcass of the goat after discarded dates supplementation in all groups (mg FA/100-gram sample).

Item	Treatments			SEM	*p*-Value
	Control	D1	D2		
Myristic acid	0.14	0.13	0.15	0.011	0.778
Pentacyclic acid	0.26	0.25	0.24	0.004	0.183
Palmitic acid	2.02	1.89	2.05	0.051	0.455
Margaric acid	0.15	0.16	0.15	0.003	0.234
Stearic acid	1.55	1.57	1.65	0.033	0.704
Arachidic acid	0.012	0.013	0.014	0.001	0.151
Behenic acid	0.01	0.01	0.01	0.001	0.319
Lignoceric acid	0.0135	0.013	0.012	0.003	0.331
Palmitoleic acid	1.84 ^a^	1.67 ^b^	1.55 ^c^	0.025	<0.001
Octadecenoic acid	1.98 ^c^	2.23 ^b^	2.33 ^a^	0.032	<0.001
Linoleic acid	3.58 ^a^	3.56 ^a^	3.47 ^b^	0.012	<0.001
Eicosatetraenoic acid	0.01	0.01	0.01	0.001	0.122
a-linolenic acid	0.25	0.23	0.23	0.003	0.127
Eicosapentaenoic acid	0.01	0.01	0.01	0.001	0.234
Docosapentaenoic acid	0.021	0.022	0.024	0.001	0.479
Rumenic acid	0.024 ^c^	0.039 ^b^	0.052 ^a^	0.001	<0.001
t10,c12-2CLA	0.165	0.166	0.168	0.001	0.944
∑SFA	4.02	3.93	4.16	0.051	0.073
∑MUFA	3.82	3.90	3.88	0.025	0.142
∑PUFA	3.86	3.86	3.77	0.012	0.067
n-3 PUFA	0.281	0.26	0.26	0.003	0.067
Total UFA (MUFA + PUFA)	7.68	7.76	7.65	0.037	0.210
n-6/n-3 Ratio	12.78	13.63	13.18	0.158	0.114

D1 = 10% discarded dates, D2 = 10% solid-state-fermented discarded dates. Rumenic acid (c9,t11-CLA) is a conjugated linoleic acid isomer. SFA = saturated fatty acid, MUFA = monounsaturated fatty acid, PUFA = polyunsaturated fatty acid, SEM = standard error of mean. ^a^, ^b^, ^c^ values within the same row with different superscript letters are significantly different (*p* < 0.05). n = 8 per treatment group.

**Table 8:** Amino acid levels in the carcass of the goat after the supplementation of discarded dates in all groups (mg/g protein).

Item	Treatments			SEM	*p*-Value
	Control	D1	D2		
Lys	15.16 ^c^	15.38 ^b^	16.82 ^a^	0.156	<0.001
Phe	7.77	7.81	7.86	0.017	0.101
Met	5.02 ^b^	5.08 ^b^	5.67 ^a^	0.064	<0.001
Thr	8.01 ^b^	8.04 ^b^	8.57 ^a^	0.056	<0.001
Ile	7.60 ^c^	7.87 ^b^	8.10 ^a^	0.043	<0.001
Leu	14.46 ^c^	15.12 ^b^	15.72 ^a^	0.108	<0.001
Val	6.98 ^c^	7.32 ^b^	7.62 ^a^	0.056	<0.001
Arg	12.08	12.05	12.07	0.017	0.773
His	6.05	6.06	6.07	0.006	0.510
Cys	1.64	1.65	1.64	0.005	0.549
Tyr	4.34	4.39	4.34	0.008	0.008
Asp	15.10	15.05	15.08	0.012	0.267
Ser	7.05	7.05	7.04	0.008	0.963
Glu	28.32 ^a^	28.12 ^b^	28.10 ^b^	0.026	<0.001
Gly	8.08	8.08	8.10	0.013	0.816
Ala	8.93	9.07	9.07	0.035	0.165

D1 = 10% discarded dates, D2 = 10% solid-state-fermented discarded dates, SEM = standard error of mean. ^a^, ^b^, ^c^ values within the same row with different superscript letters are significantly different (*p* < 0.05). n = 8 per treatment group.

## Data Availability

The original contributions presented in this study are included in the article. Further inquiries can be directed to the corresponding authors.

## Supplementary Materials

The following supporting information can be downloaded at https://www.mdpi.com/article/10.3390/vetsci13070641/s1.
