# Supplementary material for: Solid-State Fermented Discarded Dates as a Functional Feed Ingredient: Effects on Meat Quality, Fatty Acid Profile, and Essential Amino Acid Composition
Source: Vet Sci. 2026 Jun 30;13(7):641. doi: 10.3390/vetsci13070641 (PMC13431468; doi:10.3390/vetsci13070641)
Supplement: Supplementary file 1 [file vetsci-13-00641-s001.zip › vetsci-4397438-supplementary.pdf]

Supplementary File S1

Solid-State Fermented Discarded Dates as a Functional Feed Ingredient: Effects on Meat Quality, Fatty Acid Profile, and Essential Amino Acid Composition

Ali Mujtaba Shah [1,†], Dongxu Xia [2,†], Wence Wang [1], Yuan Yuan [3], Ali Raza Shah [4], Ali Mustafa Shah [5], Nazir Ahmed Khan [6], Weijie Pan [2], Wei Shi [2], Guoqiang Chen [1], Fu Yang [1], Hongxia Zhao [2,*] and Qingyun Cao [1,*]

[1] State Key Laboratory of Swine and Poultry Breeding Industry, College of Animal Science, South China Agricultural University, Guangzhou 510642, China; alimujtabashah@sbbuvas.edu.pk (A.M.S.); wangwence@scau.edu.cn (W.W.); 1016818229@qq.com (G.C.); 18156908253@163.com (F.Y.)

[2] Inner Mongolia Key Laboratory of Veterinary Fundamentals and Disease Prevention and Control for Herbivorous Livestock, College of Veterinary Medicine, Inner Mongolia Agricultural University, Hohhot, 010018, China; xia19861608693@163.com (D.X.); pwj1976933477@163.com (W.P.); hongyuan345@gmail.com (W.S.)

[3] Chongqing Three Gorges Vocational College, Chongqing 404100, China; 2008060033@cqsxzy.edu.cn

[4] Khairpur College of Agriculture and Management Sciences, Sindh Agriculture University, Tandojam 07005, Pakistan; arshah@sau.edu.pk

[5] Benazir Bhutto Shaheed University of Technology and Skill Development, Khairpur Mirs 66020, Pakistan; mustafa@bbsutsd.edu.pk

[6] Department of Animal Nutrition, The University of Agriculture, Peshawar 25130, Khyber Pakhtunkhwa, Pakistan; nazir.khan@aup.edu.pk

[*] Correspondence: zhaohongxia@imau.edu.cn (H.Z.); qycao@scau.edu.cn (Q.C.)

[†] These authors contributed equally to this work.

*Corresponding author: Email address: zhaohongxia@imau.edu.cn and qycao@scau.edu.cn

A detailed protocol of Dates solid-state fermentation

Fresh, pitted DD were received from the local market in Pakistan and transported to the experimental area. The dates were mechanically chopped (5-10 mm) using a sterile food processor. These chopped dates were mixed with 10% wheat bran in a stainless-steel mixer, and distilled water was added to achieve the required 50-51% moisture. The mixture was subsequently pasteurized in a steam chamber at 85-90 °C for 30 minutes to decrease the background microbial load while preserving heat-labile nutrients. While thermal processing can affect phenolic compounds, the mild heat treatment (85-90°C, 30 min) was selected to reduce background microbial load while minimizing degradation of heat-sensitive bioactive compounds. Subsequent SSF further enhanced phenolic content, consistent with previous reports that fermentation can compensate for moderate thermal losses through bioconversion of bound phenolics [16]. After cooling at 35-40 °C in a laminar flow hood, the substrate was aseptically inoculated with a defined mixed culture suspension containing an equal proportion of *Aspergillus oryzae* (MTCC 1846), *Saccharomyces cerevisiae* (MTCC 170), and *Lactobacillus plantarum* (MTCC 1407). The inoculum was prepared by suspending pre-cultured spores and cells in 0.1% peptone water to a final concentration of 10^8 CFU/g for each microorganism and applied uniformly at 2% (v/w) of the substrate weight, followed by thorough manual mixing using sterile gloves to ensure even distribution. The inoculated substrate was spread into a 5–8 cm thick layer in perforated polypropylene trays, covered with triple-layer sterile muslin cloth to allow gaseous exchange while preventing contamination, and incubated in a temperature-controlled environmental chamber (Memmert, Germany) maintained at 30 ± 1°C and 70–71% relative humidity for 72 h. The 72-h fermentation period was selected based on preliminary optimization experiments conducted over a 48-96 h time course with sampling at 12 h intervals (Supplementary Table S1).

This duration represented the optimal balance for maximizing both CP yield (which peaked at 72 h with a 353% increase over raw dates) and total phenolic content (which increased from 4.7 to 6.9 mg GA/g) while maintaining pH at 4.2-4.5 and ensuring microbial viability (8.5 log CFU/g for lactic acid bacteria). The termination criteria included a stable pH drop to 4.2-4.5, a dominant whitish mycelial mat, and a characteristic, acidic, fruity aroma. To promote homogeneous growth and prevent localized overheating, the fermenting mass was manually turned under aseptic conditions at 24 h intervals. The fermented product was then transferred to a hot-air oven and dried at 50 ± 2°C for 24 h to a final moisture content of ≤12%, ground using a hammer mill to pass through a 3-mm sieve, vacuum-packed in metallized polyethylene bags, and stored at ambient temperature (25–30°C) in the dark until used in feeding trials. Proximate composition, microbial load, and mycotoxin safety (aflatoxin B1, ochratoxin) were analyzed before animal supplementation.

Table S1: Optimization of solid-state fermentation parameters for discarded dates over 48-96 hours

Time (h)	CP (%)	Total Phenolics (mg GAE/g)	pH	Lactic Acid Bacteria (log CFU/g)	Yeast (log CFU/g)
0	6.8	4.7	5.8	3.2	2.1
12	7.1	4.9	5.6	4.5	3.8
24	7.8	5.3	5.1	6.2	5.1
36	8.4	5.8	4.8	7.5	6.3
48	8.8	6.3	4.6	8.1	7.2
60	9	6.6	4.5	8.4	7.6
**72**	**9.2**	**6.9**	**4.4**	**8.5**	**7.9**
84	9.1	6.9	4.3	8.3	7.7
96	8.9	6.8	4.2	8	7.4

CP, crude protein; GAE, gallic acid equivalent; CFU, colony-forming units.
